# Supplementary material for: Association between EPHA5 methylation status in peripheral blood leukocytes and the risk and prognosis of gastric cancer
Source: PeerJ. 2022 Sep 21;10:e13774. doi: 10.7717/peerj.13774 (PMC9508887; doi:10.7717/peerj.13774)
Supplement: Supplemental Information 1 [file peerj-10-13774-s001.docx]

**Table S1.** Associations between environmental factors and GC risk

| Environmental factors |  | Case (%) | Control (%) | Crude OR (95% CI) | *P* | OR^a^ (95% CI) | *P* | OR^b^ (95% CI) | *P* |
| --- | --- | --- | --- | --- | --- | --- | --- | --- | --- |
| Alcohol consumption | No | 153 (41.8) | 191 (51.1) | 1.000 |  | 1.000 |  | 1.000 |  |
|  | Yes | 213 (58.2) | 183 (48.9) | 1.469 (1.098-1.965) | 0.010 | 1.821 (1.251-2.651) | 0.002 | 1.515 (1.114-2.060) | 0.008 |
| Bean products (times/week) | ≤2 | 195 (53.3) | 159 (42.5) | 1.000 |  | 1.000 |  | 1.000 |  |
|  | >2 | 171 (46.7) | 215 (57.5) | 0.643 (0.480-0.861) | 0.003 | 0.653 (0.481-0.888) | 0.007 | 0.654 (0.482-0.888) | 0.006 |
| Beef and mutton (g/week) | <250 | 327 (89.3) | 299 (79.9) | 1.000 |  | 1.000 |  | 1.000 |  |
|  | ≥250 | 39 (10.7) | 75 (20.1) | 0.480 (0.315-0.729) | 0.001 | 0.504 (0.322-0.789) | 0.003 | 0.519 (0.334-0.807) | 0.004 |
| Chicken (g/week) | <250 | 319 (87.2) | 302 (80.7) | 1.000 |  | 1.000 |  | 1.000 |  |
|  | ≥250 | 47 (12.8) | 72 (19.3) | 0.623 (0.414-0.937) | 0.023 | 0.736 (0.482-1.125) | 0.157 | 0.739 (0.484-1.127) | 0.160 |
| Regular diet | No | 128 (35.0) | 57 (15.2) | 1.000 |  | 1.000 |  | 1.000 |  |
|  | Yes | 238 (65.0) | 317 (84.8) | 0.335 (0.235-0.479) | <0.001 | 0.352 (0.241-0.515) | <0.001 | 0.372 (0.257-0.538) | <0.001 |
| Eating speed | No | 186 (50.8) | 210 (56.1) | 1.000 |  | 1.000 |  | 1.000 |  |
|  | Yes | 180 (49.2) | 164 (43.9) | 1.234 (0.923-1.650) | 0.157 | 1.474 (1.074-2.024) | 0.016 | 1.457 (1.069-1.986) | 0.017 |
| Egg (g/week) | ≤6 | 285 (77.9) | 254 (67.9) | 1.000 |  | 1.000 |  | 1.000 |  |
|  | ≥7 | 81 (22.1) | 120 (32.1) | 0.596 (0.427-0.833) | 0.002 | 0.624 (0.438-0.889) | 0.009 | 0.630 (0.444-0.895) | 0.010 |
| Food left overnight (times/week) | <1 | 73 (19.9) | 117 (31.3) | 1.000 |  | 1.000 |  | 1.000 |  |
|  | ≥1 | 293 (80.1) | 257 (68.7) | 1.850 (1.310-2.613) | <0.001 | 2.026 (1.402-2.928) | <0.001 | 2.009 (1.393-2.897) | <0.001 |
| Freshwater fish (times/week) | <1 | 203 (55.5) | 318 (85.0) | 1.000 |  | 1.000 |  | 1.000 |  |
|  | ≥1 | 163 (44.5) | 56 (15.0) | 4.568 (3.199-6.524) | <0.001 | 5.023 (3.445-7.326) | <0.001 | 4.961 (3.409-7.218) | <0.001 |
| Fried food (times/week) | <1 | 172 (47.0) | 212 (56.7) | 1.000 |  | 1.000 |  | 1.000 |  |
|  | ≥1 | 194 (53.0) | 162 (43.3) | 1.478 (1.103-1.980) | 0.009 | 1.591 (1.160-2.182) | 0.004 | 1.555 (1.143-2.114) | 0.005 |
| Fruits (g/week) | <1000 | 222 (60.7) | 218 (58.3) | 1.000 |  | 1.000 |  | 1.000 |  |
|  | ≥1000 | 144 (39.3) | 156 (41.7) | 0.915 (0.681-1.229) | 0.553 | 0.906 (0.664-1.237) | 0.536 | 0.911 (0.669-1.241) | 0.555 |
| Garlic (times/week) | <1 | 287 (78.4) | 197 (52.7) | 1.000 |  | 1.000 |  | 1.000 |  |
|  | ≥1 | 79 (21.6) | 177 (47.3) | 0.304 (0.220-0.420) | <0.001 | 0.286 (0.202-0.405) | <0.001 | 0.298 (0.212-0.420) | <0.001 |
| Green vegetables (g/week) | <250 | 77 (21.0) | 24 (6.4) | 1.000 |  | 1.000 |  | 1.000 |  |
|  | ≥250 | 289 (79.0) | 350 (93.6) | 0.260 (0.160-0.423) | <0.001 | 0.272 (0.164-0.452) | <0.001 | 0.273 (0.165-0.452) | <0.001 |
| Hot food | No | 196 (53.6) | 201 (53.7) | 1.000 |  | 1.000 |  | 1.000 |  |
|  | Yes | 170 (46.4) | 173 (46.3) | 1.009 (0.754-1.350) | 0.952 | 0.982 (0.721-1.338) | 0.909 | 0.984 (0.724-1.337) | 0.918 |
| Marine product (times/week) | <1 | 320 (87.4) | 329 (88.0) | 1.000 |  | 1.000 |  | 1.000 |  |
|  | ≥1 | 46 (12.6) | 45 (12.0) | 1.034 (0.660-1.622) | 0.883 | 0.979 (0.608-1.576) | 0.930 | 0.990 (0.620-1.582) | 0.967 |
| Pork (g/week) | <250 | 138 (37.7) | 154 (41.2) | 1.000 |  | 1.000 |  | 1.000 |  |
|  | ≥250 | 228 (62.3) | 220 (58.8) | 1.154 (0.855-1.557) | 0.349 | 1.317 (0.951-1.825) | 0.098 | 1.301 (0.950-1.783) | 0.101 |
| Refrigerated food | No | 139 (38.0) | 72 (19.3) | 1.000 |  | 1.000 |  | 1.000 |  |
|  | Yes | 227 (62.0) | 302 (80.7) | 0.395 (0.282-0.552) | <0.001 | 0.385 (0.271-0.547) | <0.001 | 0.389 (0.274-0.552) | <0.001 |
| Salted food | No | 164 (44.8) | 243 (65.0) | 1.000 |  | 1.000 |  | 1.000 |  |
|  | Yes | 202 (55.2) | 131 (35..0) | 2.262 (1.680-3.044) | <0.001 | 2.330 (1.701-3.191) | <0.001 | 2.311 (1.691-3.159) | <0.001 |
| Tea | No | 266 (72.7) | 248 (66.3) | 1.000 |  | 1.000 |  | 1.000 |  |
|  | Yes | 100 (27.3) | 126 (33.7) | 0.741 (0.540-1.017) | 0.064 | 0.769 (0.545-1.086) | 0.136 | 0.787 (0.565-1.097) | 0.158 |
| Water | River-water and well-water | 104 (28.4) | 78 (20.9) | 1.000 |  | 1.000 |  | 1.000 |  |
|  | Tap water and mineral-water | 262 (71.6) | 296 (79.1) | 0.665 (0.472-0.937) | 0.020 | 0.654 (0.452-0.945) | 0.024 | 0.669 (0.468-0.958) | 0.028 |
| Dairy products | <1 | 142 (38.8) | 213 (57.0) | 1.000 |  | 1.000 |  | 1.000 |  |
|  | ≥1 | 224 (61.2) | 161 (43.0) | 2.076 (1.547-2.785) | <0.001 | 2.049 (1.503-2.793) | <0.001 | 2.047 (1.503-2.787) | <0.001 |
| *H. pylori* infection | Negative | 136 (37.2) | 193 (51.6) | 1.000 |  | 1.000 |  | 1.000 |  |
|  | Positive | 230 (62.8) | 181 (48.4) | 1.795 (1.327-2.429) | <0.001 | 1.884 (1.368-2.593) | <0.001 | 1.874(1.363-2.576) | <0.001 |
| Smoking | No | 138 (37.7) | 181 (48.4) | 1.000 |  | 1.000 |  | 1.000 |  |
|  | Yes | 228 (62.3) | 193 (51.6) | 1.537 (1.144-2.066) | 0.004 | 1.855 (1.308-2.630) | 0.001 | 1.655 (1.211-2.262) | 0.002 |

**Notes.**

OR was calculated by Logistic regression analysis. ^a^ Adjusted to age, sex, BMI, income and family history of gastric cancer. ^b^ Adjusted to propensity score of age, sex, BMI, income and family history of gastric cancer.

**Table S2.** Multivariate analysis of the associations between environmental factors and GC risk

| Variable |  | *β-coefficient* | SE | *P* | OR (95% CI) |
| --- | --- | --- | --- | --- | --- |
| *H. pylori* infection | Negative |  |  |  | 1.000 |
|  | Positive | 0.754 | 0.209 | <0.001 | 2.125 (1.411-3.200) |
| Regular diet | No |  |  |  | 1.000 |
|  | Yes | -1.384 | 0.251 | <0.001 | 0.251 (0.153-0.410) |
| Salted food | No |  |  |  | 1.000 |
|  | Yes | 0.938 | 0.211 | <0.001 | 2.555 (1.688-3.866) |
| Green vegetables (g/week) | <250 |  |  |  | 1.000 |
|  | ≥250 | -1.388 | 0.335 | <0.001 | 0.250 (0.130-0.481) |
| Garlic (times/week) | <1 |  |  |  | 1.000 |
|  | ≥1 | -1.500 | 0.234 | <0.001 | 0.223 (0.141-0.353) |
| Beef and mutton (g/week) | <250 |  |  |  | 1.000 |
|  | ≥250 | -0.839 | 0.323 | 0.009 | 0.432 (0.230-0.814) |
| Freshwater fish (times/week) | <1 |  |  |  | 1.000 |
|  | ≥1 | 1.903 | 0.246 | <0.001 | 6.707 (4.145-10.855) |
| Dairy products | <1 |  |  |  | 1.000 |
|  | ≥1 | 1.338 | 0.233 | <0.001 | 3.810 (2.411-6.020) |
| Fried food (times/week) | <1 |  |  |  | 1.000 |
|  | ≥1 | 0.601 | 0.212 | 0.005 | 1.824 (1.204-2.761) |
| Refrigerated food | No |  |  |  | 1.000 |
|  | Yes | -1.511 | 0.252 | <0.001 | 0.221 (0.135-0.361) |
| Alcohol consumption | No |  |  |  | 1.000 |
|  | Yes | 0.726 | 0.255 | 0.004 | 2.067 (1.253-3.410) |
| Water | River-water and well-water |  |  |  | 1.000 |
|  | Tap water and mineral-water | -0.817 | 0.250 | 0.001 | 0.442 (0.271-0.720) |
| Food left overnight (times/week) | <1 |  |  |  | 1.000 |
|  | ≥1 | 1.018 | 0.253 | <0.001 | 2.768 (1.686-4.546) |
| Sex | Male |  |  |  | 1.000 |
|  | Female | 0.765 | 0.301 | 0.011 | 2.150 (1.192-3.876) |
| Age | <60 |  |  |  | 1.000 |
|  | ≥60 | 0.070 | 0.217 | 0.746 | 1.073 (0.701-1.641) |
| BMI (kg/m^2^) | <23.00 |  |  |  | 1.000 |
|  | ≥23.00 | -0.870 | 0.212 | <0.001 | 0.419 (0.276-0.635) |
| Monthly income (Dollars/ Per capita) | <150 |  |  |  | 1.000 |
|  | ≥150 | 0.704 | 0.222 | 0.002 | 2.021 (1.307-3.125) |
| Family history of gastric cancer | No |  |  |  | 1.000 |
|  | Yes | 2.188 | 0.446 | <0.001 | 8.913 (3.721-21.349) |
| constant |  | -1.241 | 1.410 | 0.379 | 0.289 |

**Notes.**

Statistical differences was conducted by backward conditional selection method.

**Table S3.** Associations between environmental factors and *EPHA5* methylation

| Environmental factors |  | Pm | Nm | Crude OR (95% CI) | *P* | OR^a^ (95% CI) | *P* | OR^b^ (95% CI) | *P* |
| --- | --- | --- | --- | --- | --- | --- | --- | --- | --- |
| Alcohol consumption | No | 143 (47.2) | 201 (46.0) | 1.000 |  | 1.000 |  | 1.000 |  |
|  | Yes | 160 (52.8) | 236 (54.0) | 0.953 (0.708-1.282) | 0.750 | 1.148 (0.794-1.659) | 0.463 | 0.950 (0.705-1.280) | 0.736 |
| Bean products (times/week) | ≤2 | 137 (45.2) | 217 (49.7) | 1.000 |  | 1.000 |  | 1.000 |  |
|  | >2 | 166 (54.8) | 220 (50.3) | 1.193 (0.887-1.604) | 0.242 | 1.232 (0.910-1.668) | 0.178 | 1.217 (0.904-1.638) | 0.195 |
| Beef and mutton (g/week) | <250 | 261 (86.1) | 365 (83.5) | 1.000 |  | 1.000 |  | 1.000 |  |
|  | ≥250 | 42 (13.9) | 72 (16.5) | 0.830 (0.543-1.269) | 0.390 | 0.949 (0.612-1.471) | 0.815 | 0.862 (0.563-1.319) | 0.493 |
| Chicken (g/week) | <250 | 266 (87.8) | 355 (81.2) | 1.000 |  | 1.000 |  | 1.000 |  |
|  | ≥250 | 37 (12.2) | 82 (18.8) | 0.595 (0.388-0.915) | 0.018 | 0.619 (0.396-0.967) | 0.035 | 0.627 (0.406-0.967) | 0.035 |
| Regular diet | No | 73 (24.1) | 112 (25.6) | 1.000 |  | 1.000 |  | 1.000 |  |
|  | Yes | 230 (75.9) | 325 (74.4) | 1.083 (0.770-1.524) | 0.646 | 1.018 (0.710-1.460) | 0.924 | 1.152 (0.814-1.630) | 0.424 |
| Eating speed | No | 160 (52.8) | 236 (54.0) | 1.000 |  | 1.000 |  | 1.000 |  |
|  | Yes | 143 (47.2) | 201 (46.0) | 1.045 (0.777-1.406) | 0.771 | 1.225 (0.896-1.675) | 0.203 | 1.086 (0.805-1.466) | 0.588 |
| Egg (g/week) | ≤6 | 223 (73.6) | 316 (72.3) | 1.000 |  | 1.000 |  | 1.000 |  |
|  | ≥7 | 80 (26.4) | 121 (27.7) | 0.940 (0.672-1.313) | 0.715 | 0.951 (0.673-1.343) | 0.774 | 0.966 (0.689-1.354) | 0.841 |
| Food left overnight (times/week) | <1 | 72 (23.8) | 118 (27.0) | 1.000 |  | 1.000 |  | 1.000 |  |
|  | ≥1 | 231 (76.2) | 319 (73.0) | 1.177 (0.838-1.654) | 0.347 | 1.228 (0.867-1.740) | 0.247 | 1.185 (0.841-1.668) | 0.332 |
| Freshwater fish (times/week) | <1 | 204 (67.3) | 318 (72.8) | 1.000 |  | 1.000 |  | 1.000 |  |
|  | ≥1 | 99 (32.7) | 119 (27.2) | 1.299 (0.941-1.795) | 0.112 | 1.323 (0.950-1.842) | 0.097 | 1.275 (0.921-1.765) | 0.143 |
| Fried food (times/week) | <1 | 149 (49.2) | 234 (53.5) | 1.000 |  | 1.000 |  | 1.000 |  |
|  | ≥1 | 154 (50.8) | 203 (46.5) | 1.195 (0.888-1.607) | 0.239 | 1.353 (0.992-1.848) | 0.057 | 1.201 (0.892-1.616) | 0.228 |
| Fruits (g/week) | <1000 | 175 (57.8) | 265 (60.6) | 1.000 |  | 1.000 |  | 1.000 |  |
|  | ≥1000 | 128 (42.2) | 172 (39.4) | 1.119 (0.829-1.511) | 0.462 | 1.184 (0.871-1.610) | 0.280 | 1.120 (0.829-1.514) | 0.460 |
| Garlic (times/week) | <1 | 200 (66.0) | 284 (65.0) | 1.000 |  | 1.000 |  | 1.000 |  |
|  | ≥1 | 103 (34.0) | 153 (35.0) | 0.964 (0.705-1.318) | 0.818 | 1.071 (0.775-1.481) | 0.676 | 0.990 (0.722-1.356) | 0.948 |
| Green vegetables (g/week) | <250 | 60 (19.8) | 42 (9.6) | 1.000 |  | 1.000 |  | 1.000 |  |
|  | ≥250 | 243 (80.2) | 395 (90.4) | 0.428 (0.279-0.658) | <0.001 | 0.446 (0.287-0.694) | <0.001 | 0.446 (0.289-0.687) | <0.001 |
| Hot food | No | 159 (52.5) | 237 (54.2) | 1.000 |  | 1.000 |  | 1.000 |  |
|  | Yes | 144 (47.5) | 200 (45.8) | 1.071 (0.797-1.441) | 0.648 | 1.097 (0.809-1.487) | 0.550 | 1.064 (0.790-1.433) | 0.684 |
| Marine product (times/week) | <1 | 262 (86.5) | 387 (88.6) | 1.000 |  | 1.000 |  | 1.000 |  |
|  | ≥1 | 41 (13.5) | 50 (11.4) | 1.212 (0.771-1.905) | 0.405 | 1.270 (0.795-2.027) | 0.317 | 1.194 (0.759-1.880) | 0.443 |
| Pork (g/week) | <250 | 128 (42.2) | 164 (37.5) | 1.000 |  | 1.000 |  | 1.000 |  |
|  | ≥250 | 175 (57.8) | 273 (62.5) | 0.829 (0.614-1.120) | 0.223 | 0.951 (0.694-1.303) | 0.753 | 0.850 (0.629-1.151) | 0.293 |
| Refrigerated food | No | 89 (29.4) | 122 (27.9) | 1.000 |  | 1.000 |  | 1.000 |  |
|  | Yes | 214 (70.6) | 315 (72.1) | 0.925 (0.666-1.284) | 0.640 | 0.942 (0.673-1.317) | 0.725 | 0.941 (0.678-1.308) | 0.719 |
| Salted food | No | 160 (52.8) | 247 (56.5) | 1.000 |  | 1.000 |  | 1.000 |  |
|  | Yes | 143 (47.2) | 190 (43.5) | 1.150 (0.856-1.545) | 0.354 | 1.204 (0.890-1.629) | 0.228 | 1.133 (0.842-1.524) | 0.411 |
| Tea | No | 213 (70.3) | 301 (68.9) | 1.000 |  | 1.000 |  | 1.000 |  |
|  | Yes | 90 (29.7) | 136 (31.1) | 0.934 (0.678-1.286) | 0.675 | 1.042 (0.742-1.462) | 0.814 | 0.956 (0.692-1.319) | 0.783 |
| Water | River-water and well-water | 71 (23.4) | 111 (25.4) | 1.000 |  | 1.000 |  | 1.000 |  |
|  | Tap water and mineral-water | 232 (76.6) | 326 (74.6) | 1.128 (0.797-1.597) | 0.496 | 1.162 (0.809-1.668) | 0.417 | 1.144 (0.808-1.621) | 0.449 |
| Dairy products | <1 | 140 (46.2) | 215 (49.2) | 1.000 |  | 1.000 |  | 1.000 |  |
|  | ≥1 | 163 (53.8) | 222 (50.8) | 1.135 (0.843-1.528) | 0.403 | 1.090 (0.805-1.477) | 0.576 | 1.109 (0.822-1.496) | 0.497 |
| *H. pylori* infection | Negative | 134 (44.2) | 195 (44.6) | 1.000 |  | 1.000 |  | 1.000 |  |
|  | Positive | 169 (55.8) | 242 (55.4) | 1.020 (0.758-1.374) | 0.894 | 1.011 (0.746-1.371) | 0.944 | 1.016 (0.754-1.370) | 0.915 |
| Smoking | No | 116 (38.3) | 204 (46.7) | 1.000 |  | 1.000 |  | 1.000 |  |
|  | Yes | 187 (61.7) | 233 (53.3) | 1.415 (1.047-1.913) | 0.024 | 1.757 (1.242-2.485) | 0.001 | 1.432 (1.058-1.939) | 0.020 |

**Notes.**

Nm, negative methylation; Pm, positive methylation

OR was calculated by Logistic regression analysis. ^a^ Adjusted to age, sex, BMI, income and family history of gastric cancer. ^b^ Adjusted to propensity score of age, sex, BMI, income and family history of gastric cancer.

**Table S4.** Effects of the combination and interaction between environmental factors and *EPHA5* methylation status on the risk of GC

| Environmental factors |  | *EPHA5* methylation status | | | | | | | |
| --- | --- | --- | --- | --- | --- | --- | --- | --- | --- |
|  |  | Nm | |  | Pm | |  | Interactions | |
|  |  | OR_c_^a^ (95% CI) | *P* |  | OR_c_^a^ (95% CI) | *P* |  | OR_i_^a^ (95% CI) | *P* |
| Alcohol consumption | No | 1.000 |  |  | 1.966 (1.241-3.115) | 0.004 |  | 1.000 |  |
|  | Yes | 1.581 (1.057-2.365) | 0.026 |  | 2.893 (1.844-4.539) | <0.001 |  | 0.931 (0.494-1.751) | 0.823 |
| Beef and mutton (g/week) | <250 | 1.000 |  |  | 1.913 (1.360-2.690) | <0.001 |  | 1.000 |  |
|  | ≥250 | 0.555 (0.308-0.998) | 0.049 |  | 0.906 (0.461-1.778) | 0.773 |  | 0.853 (0.341-2.135) | 0.735 |
| Regular diet | No | 1.000 |  |  | 4.714 (2.151-10.329) | <0.001 |  | 1.000 |  |
|  | Yes | 0.505 (0.320-0.798) | 0.003 |  | 0.804 (0.499-1.296) | 0.370 |  | 0.338 (0.142-0.803) | 0.014 |
| Food left overnight (times/week) | <1 | 1.000 |  |  | 1.805 (0.948-3.437) | 0.072 |  | 1.000 |  |
|  | ≥1 | 1.952 (1.217-3.133) | 0.006 |  | 3.649 (2.215-6.013) | <0.001 |  | 1.036 (0.492-2.181) | 0.926 |
| Freshwater fish (times/week) | <1 | 1.000 |  |  | 1.765 (1.203-2.591) | 0.004 |  | 1.000 |  |
|  | ≥1 | 4.586 (2.849-7.381) | <0.001 |  | 9.750 (5.376-17.684) | <0.001 |  | 1.204 (0.552-2.628) | 0.640 |
| Fried food (times/week) | <1 | 1.000 |  |  | 1.938 (1.248-3.010) | 0.003 |  | 1.000 |  |
|  | ≥1 | 1.590 (1.065-2.374) | 0.023 |  | 2.819 (1.807-4.399) | <0.001 |  | 0.915 (0.486-1.722) | 0.783 |
| Garlic (times/week) | <1 | 1.000 |  |  | 1.798 (1.213-2.665) | 0.004 |  | 1.000 |  |
|  | ≥1 | 0.255 (0.160-0.408) | <0.001 |  | 0.607 (0.375-0.983) | 0.042 |  | 1.322 (0.656-2.663) | 0.435 |
| Green vegetables (g/week) | <250 | 1.000 |  |  | 3.822 (1.405-10.398) | 0.009 |  | 1.000 |  |
|  | ≥250 | 0.453 (0.228-0.901) | 0.024 |  | 0.704 (0.348-1.426) | 0.330 |  | 0.407 (0.141-1.173) | 0.096 |
| Refrigerated food | No | 1.000 |  |  | 1.646 (0.883-3.067) | 0.117 |  | 1.000 |  |
|  | Yes | 0.357 (0.228-0.560) | <0.001 |  | 0.715 (0.445-1.147) | 0.164 |  | 1.215 (0.585-2.523) | 0.601 |
| Salted food | No | 1.000 |  |  | 2.292 (1.490-3.527) | <0.001 |  | 1.000 |  |
|  | Yes | 2.775 (1.840-4.187) | <0.001 |  | 4.018 (2.538-6.362) | <0.001 |  | 0.632 (0.332-1.201) | 0.161 |
| Dairy products | <1 | 1.000 |  |  | 1.373 (0.869-2.169) | 0.174 |  | 1.000 |  |
|  | ≥1 | 1.606 (1.076-2.398) | 0.020 |  | 4.024 (2.550-6.348) | <0.001 |  | 1.824 (0.959-3.470) | 0.067 |
| Water | River-water and well-water | 1.000 |  |  | 1.995 (1.040-3.825) | 0.038 |  | 1.000 |  |
|  | Tap water and mineral-water | 0.667 (0.421-1.055) | 0.083 |  | 1.249 (0.772-2.022) | 0.366 |  | 0.939 (0.446-1.975) | 0.868 |
| *H. pylori* infection | Negative | 1.000 |  |  | 2.244 (1.384-3.637) | 0.001 |  | 1.000 |  |
|  | Positive | 2.146 (1.409-3.269) | <0.001 |  | 3.543 (2.233-5.621) | <0.001 |  | 0.736 (0.385-1.404) | 0.352 |

**Notes.**

Nm, negative methylation; Pm, positive methylation

OR_c_ was calculated by crossover analysis. OR_i_ was calculated by multivariate logistic regression analysis with a product-term coefficient. ^a^ Adjusted for propensity score of age, sex, BMI, income and family history of gastric cancer.

**Table S5.** Associations between demographic characteristics and prognosis of GC

| Demographic characteristics |  | Case (%) | HR (95% CI) | *P* |
| --- | --- | --- | --- | --- |
| Sex | Male | 261 (75.7) | 1.000 |  |
|  | Female | 84 (24.3) | 1.217 (0.872-1.698) | 0.248 |
| Age | <60 | 194 (56.2) | 1.000 |  |
|  | ≥60 | 151 (43.8) | 1.232 (0.919-1.652) | 0.163 |
| BMI (kg/m^2^) | ≤18.50 | 41 (11.9) | 1.000 |  |
|  | 18.50-23.00 | 171 (49.6) | 0.791 (0.500-1.251) | 0.316 |
|  | ≥23.00 | 133 (38.5) | 0.681 (0.422-1.100) | 0.116 |
| Monthly income (Dollars/ Per capita) | <150 | 124 (35.9) | 1.000 |  |
|  | ≥150 | 221 (64.1) | 1.057 (0.772-1.446) | 0.731 |
| Family history of gastric cancer | No | 301 (87.2) | 1.000 |  |
|  | Yes | 44 (12.8) | 1.176 (0.766-1.805) | 0.459 |
| *H. pylori* infection | Negative | 126 (36.5) | 1.000 |  |
|  | Positive | 219 (63.5) | 0.943 (0.688-1.292) | 0.713 |
| Gastric ulcer | No | 66 (19.1) | 1.000 |  |
|  | Yes | 279 (80.9) | 1.205 (0.809-1.794) | 0.359 |
| Chronic gastritis | No | 56 (16.2) | 1.000 |  |
|  | Yes | 289 (83.8) | 1.245 (0.802-1.933) | 0.329 |

**Notes.**

HR was calculated by univariate Cox regression analysis.

**Table S6.** Multivariate analysis of GC prognosis

| Variable | | *β-coefficient* | SE | *P* | HR (95% CI) |
| --- | --- | --- | --- | --- | --- |
| Sex | Male |  |  |  | 1.000 |
|  | Female | 0.158 | 0.172 | 0.360 | 1.171 (0.835-1.641) |
| Age | <60 |  |  |  | 1.000 |
|  | ≥60 | 0.096 | 0.152 | 0.525 | 1.101 (0.818-1.483) |
| BMI (kg/m^2^) | ≤18.50 |  |  |  | 1.000 |
|  | 18.50-23.00 | -0.132 | 0.230 | 0.567 | 0.876 (0.558-1.377) |
|  | ≥23.00 | -0.231 | 0.245 | 0.346 | 0.794 (0.491-1.283) |
| Tumor size | <5cm |  |  |  | 1.000 |
|  | ≥5cm | 0.463 | 0.162 | 0.004 | 1.589 (1.158-2.181) |
| TNM stage | Ⅰ |  |  |  | 1.000 |
|  | Ⅱ | 0.452 | 0.628 | 0.472 | 1.571 (0.459-5.380) |
|  | Ⅲ | 1.172 | 0.418 | 0.005 | 3.227 (1.422-7.323) |
|  | Ⅳ | 1.868 | 0.393 | <0.001 | 6.473 (2.995-13.992) |

**Notes.**

Statistical differences was conducted by backward conditional selection method.

**Table S7.** Association between methylation status of *EPHA5* and GC prognosis by age-stratified analysis

| Methylation status | | <60 years | | | | |  | ≥60 years | | | | |
| --- | --- | --- | --- | --- | --- | --- | --- | --- | --- | --- | --- | --- |
|  |  | Case (%) | HR^a^ (95% CI) | *P* | HR^b^ (95% CI) | *P* |  | Case (%) | HR^a^ (95% CI) | *P* | HR^b^ (95% CI) | *P* |
| *EPHA5* | Nm | 111 (57.2) | 1.000 |  | 1.000 |  |  | 69 (45.7) | 1.000 |  | 1.000 |  |
|  | Pm | 83 (42.8) | 0.513 (0.326-0.807) | 0.004 | 0.645 (0.409-1.016) | 0.059 |  | 82 (54.3) | 1.663 (1.054-2.623) | 0.029 | 1.506 (0.943-2.405) | 0.087 |

**Notes.**

Nm, negative methylation; Pm, positive methylation

HR was calculated by multivariate Cox regression analysis. ^a^ Adjusted for sex, BMI, tumor size, TNM stage. ^b^ Adjusted for propensity score of all variables except age. Bonferroni correction with *P*<0.025 was considered to be statistically significant.

**Table S8.** Association between methylation status of *EPHA5* and GC prognosis by tumor size-stratified analysis

| Methylation status | | <5cm | | | | |  | ≥5cm | | | | |
| --- | --- | --- | --- | --- | --- | --- | --- | --- | --- | --- | --- | --- |
|  |  | Case (%) | HR^a^ (95% CI) | *P* | HR^b^ (95% CI) | *P* |  | Case (%) | HR^a^ (95% CI) | *P* | HR^b^ (95% CI) | *P* |
| *EPHA5* | Nm | 88 (52.1) | 1.000 |  | 1.000 |  |  | 92 (52.3) | 1.000 |  | 1.000 |  |
|  | Pm | 81 (47.9) | 1.589 (0.921-2.740) | 0.096 | 1.353 (0.786-2.330) | 0.275 |  | 84 (47.7) | 0.671 (0.450-1.000) | 0.050 | 0.689 (0.462-1.025) | 0.066 |

**Notes.**

Nm, negative methylation; Pm, positive methylation

HR was calculated by multivariate Cox regression analysis. ^a^ Adjusted for age, sex, BMI, TNM stage. ^b^ Adjusted for propensity score of all variables except tumor size. Bonferroni correction with *P*<0.025 was considered to be statistically significant.

**Table S9.** Association between methylation status of *EPHA5* and GC prognosis by sex-stratified analysis

| Methylation status | | Male | | | | |  | Female | | | | |
| --- | --- | --- | --- | --- | --- | --- | --- | --- | --- | --- | --- | --- |
|  |  | Case (%) | HR^a^ (95% CI) | *P* | HR^b^ (95% CI) | *P* |  | Case (%) | HR^a^ (95% CI) | *P* | HR^b^ (95% CI) | *P* |
| *EPHA5* | Nm | 118 (45.2) | 1.000 |  | 1.000 |  |  | 37 (44.0) | 1.000 |  | 1.000 |  |
|  | Pm | 143 (54.8) | 1.054 (0.742-1.498) | 0.767 | 1.024 (0.715-1.465) | 0.899 |  | 47 (56.0) | 0.725 (0.386-1.363) | 0.319 | 0.825 (0.400-1.701) | 0.602 |

**Notes.**

Nm, negative methylation; Pm, positive methylation

HR was calculated by multivariate Cox regression analysis. ^a^ Adjusted for age, BMI, tumor size, TNM stage. ^b^ Adjusted for propensity score of all variables except sex. Bonferroni correction with *P*<0.025 was considered to be statistically significant.

**Table S10.** Association between methylation status of *EPHA5* and GC prognosis by *H. pylori* infection-stratified analysis

| Methylation status | | *H. Pylori* Negative | | | | |  | *H. Pylori* Positive | | | | |
| --- | --- | --- | --- | --- | --- | --- | --- | --- | --- | --- | --- | --- |
|  |  | Case (%) | HR^a^ (95% CI) | *P* | HR^b^ (95% CI) | *P* |  | Case (%) | HR^a^ (95% CI) | *P* | HR^b^ (95% CI) | *P* |
| *EPHA5* | Nm | 60 (47.6) | 1.000 |  | 1.000 |  |  | 120 (54.8) | 1.000 |  | 1.000 |  |
|  | Pm | 66 (52.4) | 0.901 (0.531-1.528) | 0.698 | 0.795 (0.460-1.373) | 0.410 |  | 99 (45.2) | 0.898 (0.606-1.333) | 0.595 | 0.998 (0.673-1.480) | 0.993 |

**Notes.**

Nm, negative methylation; Pm, positive methylation

HR was calculated by multivariate Cox regression analysis. ^a^ Adjusted for age, sex, BMI, tumor size, TNM stage. ^b^ Adjusted for propensity score of all variables except *H. pylori* infection. Bonferroni correction with *P*<0.025 was considered to be statistically significant.

**Table S11.** Association between methylation status of *EPHA5* and GC prognosis by TNM stage-stratified analysis

| Methylation status | | TNM (Ⅰ+Ⅱ) | | | | |  | TNM (Ⅲ+Ⅳ) | | | | |
| --- | --- | --- | --- | --- | --- | --- | --- | --- | --- | --- | --- | --- |
|  |  | Case (%) | HR^a^ (95% CI) | *P* | HR^b^ (95% CI) | *P* |  | Case (%) | HR^a^ (95% CI) | *P* | HR^b^ (95% CI) | *P* |
| *EPHA5* | Nm | 33 (48.5) | 1.000 |  | 1.000 |  |  | 144 (52.0) | 1.000 |  | 1.000 |  |
|  | Pm | 35 (51.5) | 1.201 (0.282-5.111) | 0.802 | 1.192 (0.260-5.466) | 0.821 |  | 133 (48.0) | 0.902 (0.657-1.238) | 0.523 | 0.953 (0.693-1.311) | 0.768 |

**Notes.**

Nm, negative methylation; Pm, positive methylation

HR was calculated by multivariate Cox regression analysis. ^a^ Adjusted for age, sex, BMI, tumor size. ^b^ Adjusted for propensity score of all variables except TNM stage. Bonferroni correction with *P*<0.025 was considered to be statistically significant.
