# Supplementary material for: Association between EPHA5 methylation status in peripheral blood leukocytes and the risk and prognosis of gastric cancer
Source: PeerJ. 2022 Sep 21;10:e13774. doi: 10.7717/peerj.13774 (PMC9508887; doi:10.7717/peerj.13774)
Supplement: Supplemental Information 2 [file peerj-10-13774-s002.docx]

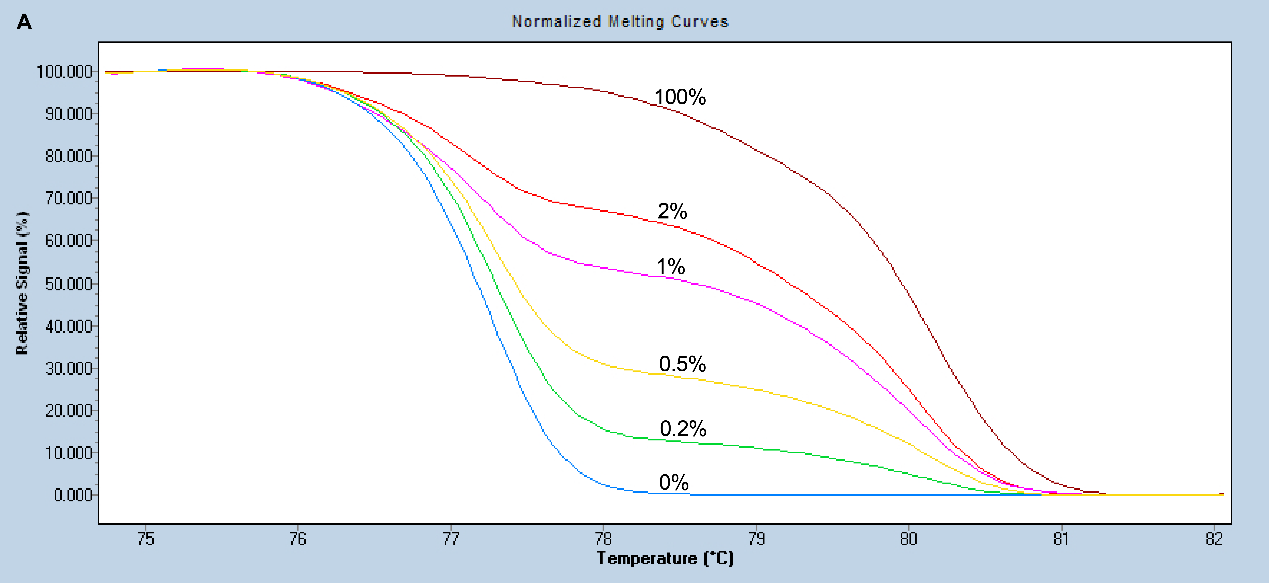


**Figure S1.** Normalized melting curves of the MS-HRM assay for *EPHA5*.


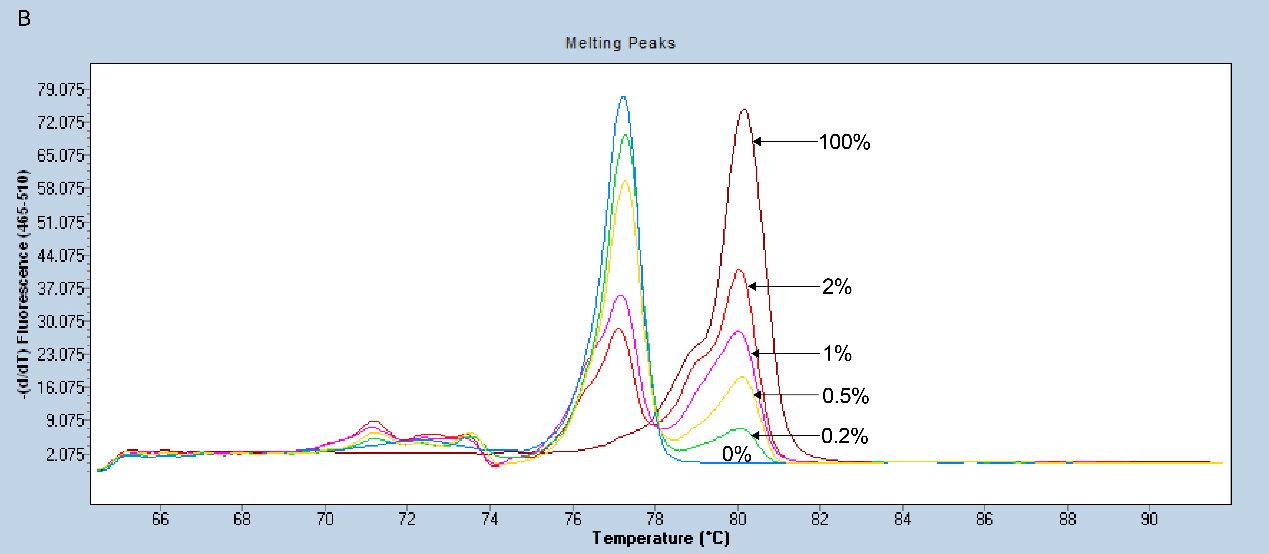


**Figure S2.** Melting peaks of the MS-HRM assay for *EPHA5*.
